# Evaluation of the implementation effect of hepatitis C medical insurance reimbursement policy in China: A RWS based on medical institutions

**DOI:** 10.3389/fpubh.2022.1072493

**Published:** 2023-01-11

**Authors:** Yiyao Liu, Liangwen Gou, Zhaoting Guo, Zhiang Wu, Qin He, Haihuan Feng, Ming Hu

**Affiliations:** ^1^West China School of Pharmacy, Sichuan University, Chengdu, China; ^2^West China Biomedical Big Data Center, West China Hospital, Sichuan University, Chengdu, China; ^3^Yeehong Business School, Shenyang Pharmaceutical University, Beijing, China; ^4^Medical Insurance Office, West China Hospital of Sichuan University, Chengdu, China

**Keywords:** hepatitis C, policy analysis, medical insurance, interrupted time series analysis, real data analysis

## Abstract

**Objectives:**

To evaluate the implementation effect of hepatitis C medical insurance reimbursement policy in China from the view of medical institutions.

**Methods:**

The electronic medical record of a top tertiary hospital in Chengdu from January 2014 to December 2020 were extracted, and the interrupted time series model was used to analyze the changes in diagnosis and treatment behavior and disease burden of hepatitis C patients after the implementation of HCV insurance reimbursement policy.

**Results:**

In terms of diagnosis and treatment, the number of visits (β2 = 19.290, *P* < 0.001) and treatments (β2 = 14.291, *P* < 0.01) increased instantaneously after the implementation of the outpatient reimbursement policy in Chengdu in 2018, and there was no significant change after the implementation of the single line payment policy for oral direct antiviral (DAA) drugs in 2019 (*P* > 0.05); in terms of medical expenses, the total treatment cost (β2 = 21439.3, *P* < 0.001), out-of-pocket expenses (β2 = 6109.44, *P* < 0.001) and drug expenses (β2 = 21889.8, *P* < 0.001) of hepatitis C patients have been significantly reduced after the implementation of the single-line payment policy.

**Conclusion:**

Hepatitis C medical insurance reimbursement policy can promote hepatitis C patients to actively seek medical treatment, promote the widespread use of DAA scheme, reduce the burden of patients, and improve the treatment efficiency of hepatitis C.

## 1. Introduction

Viral hepatitis C is an infectious disease caused by the Hepatitis C Virus (HCV), and can develop into chronice HCV infection when viremia persists for 6 months (1). Hepatitis C is endemic globally, with a worldwide Anti-HCV positivity rate of 3% according to the World Health Organization (WHO) data (2). The anti-HCV positivity rate in the general population in China is 0.43% (3), but due to the large population base in China, it is estimated that there are about 7.6 million cases of hepatitis C infection in the country (1). Chronic hepatitis C is a long-term progressive disease, and most patients are diagnosed in terminal stage of the disease, and often has already developed into cirrhosis and liver cancer (4), causing serious harm to the patient's life and health. Chronic hepatitis C also causes heavy financial burdens on patients, their families and the society. According to the results of a 2016 survey on the cost of patients with chronic HCV infection in China (5) and the statistics of the China Liver Transplant Registration, the average comprehensive treatment costs of compensated cirrhosis and de-compensated cirrhosis were 18,000 yuan and 30,000 yuan per year, respectively, and the treatment costs in the first and subsequent years of liver transplantation were 629,000 yuan and 92,000 yuan per year (1). According to the National Bureau of Statistics (http://www.stats.gov.cn/) from China, the cost of the treatment has far exceeded the per capita disposable income of Chinese residents. Once HCV progresses, it places a heavy financial burden on the patient's family.

Guidelines on chronic hepatitis C within China and abroad recommend that all patients with chronic hepatitis C (HCV-RNA positive), regardless of cirrhosis, chronic kidney disease, or extrahepatic manifestations, should receive antiviral therapy to obtain cure, thus to prevent progressions to advanced liver diseases such as cirrhosis and liver cancer, to improve long-term survival rates, to improve quality of life of patients, and to prevent HCV transmission (6–8). Direct antiviral drugs (DAAs) are a class of small molecule compounds that can directly act on an important part of HCV replication and effectively inhibit the activity of the virus (9). Clinical studies have shown that DAA drugs have achieved a sustained viral response rate (SVR) of more than 90% in HCV-infected patients with both known primary genotype and major genotype (10). In the 2018 European Society of Hepatology (EASL) HCV guidelines, interferon-free (IFN), ribavirin-free, DAA-based regimens are recommended as the preferred treatment for patients with HCV infection without cirrhosis or compensatory cirrhosis (11). In April 2017, the first DAA drug, Daclatasvir and Asunaprevir, was approved in China for the treatment of adult genotype 1b chronic hepatitis C (non-cirrhosis or compensatory cirrhosis). Since then, Ombitasvir and Dasabuvir have been listed for the treatment of genotype 1 HCV. In September 2017, Gilead Scienses' first pan genotype DAA drug to cover chronic hepatitis-SOVALDI (generic name: sofosbuvir) was approved in China through the priority review and approval process. After 2018, a number of pan genotype DAA drugs have been approved, including Ledipasvir and Sofosbuvir, Elbasvir and Grazoprevir, Sofosbuvir and Velpatasvir and Glecaprevir and Pibrentasvir. China's Hepatitis C Prevention and Control Guidelines (2019) also gave priority to recommend interferon-free DAA for treatment (6), and Sofosbuvir and Velpatasvir has also been included in the 2018 National Essential Medicines List in China.

DAA treatment have significantly improved the therapeutic effect of hepatitis C, but the availability and affordability of DAA drugs have been limited due to higher drug prices (12). Prior to 2019, no DAA drugs have been included in the national medical insurance directory, but some regions have started to explore the medical insurance payment policy of DAA drugs according to the operation of their own medical insurance funds and the status of hepatitis C prevention and treatment. The exploration included payment by capitation, payment by classification, payment by disease category, inclusion in the provincial medical insurance directory, etc. As one of the first cities in China to pilot an innovative payment model for hepatitis C, Chengdu, the capital city of Sichuan Province and the most populated city in Southwest of China, issued the Notice on Issues Related to the Implementation of Medical Insurance Classification and Payment for Hepatitis C Outpatient Medical Expenses in 2018, which included the expenses of DAA drugs and related examinations and tests into the scope of “hepatitis C” outpatient medical classification payment coverage (hereinafter referred to as the “special disease outpatient reimbursement policy”) for insured residents of Chengdu. In this policy, the cost of oral direct antiviral drugs incurred by patients in the course of treatment in designated medical institutions is allocated per head count, the upper payment limit is 43,100 yuan/person with no starting line is set. In 2019, three DAA drugs (Sofosbuvir and Velpatasvir Tablets, Ledipasvir and Sofosbuvir Tablets and Elbasvir and Grazoprevire Tablets) were included in the national medical insurance catalog through national medical insurance negotiations, and Chengdu implemented a direct payment management for these three DAA drugs, giving higher reimbursement treatment than other medical insurance Class B drugs (hereinafter referred to as the “single-line payment policy”). Whether buying drugs in hospitals or designated special pharmacies, urban employees, urban and rural residents low-end participants and urban and rural residents high-end participants would be reimbursed respectively directly from the medical insurance pooling fund, with an annual cumulative limit of 150,000 yuan/person. Whether China's innovative medical insurance policies can effectively promote the prevention and treatment of hepatitis C to achieve elimination of hepatitis C and change in the medical burden of hepatitis C patients remain to be evaluated.

Based on the real-world data of the database of a top tertiary hospital in Chengdu, this study proposes to analyze the changes in the diagnosis and treatment behavior and disease burden of hepatitis C patients before and after the implementation of the HCV medical insurance reimbursement policy from the aspects of diagnosis and treatment and medical costs, so as to provide support evidence for the prevention and treatment of hepatitis C and the formulation and improvement of medical insurance payment policies.

## 2. Methods and materials

### 2.1. Data source

The data of this study was derived from the electronic medical record of West China Hospital, a top tertiary hospital located in Chengdu and one of the largest hospitals in China. It is a designated medical institution for hepatitis C treatment, and receives the most HCV patients in southwest China. Empirical analysis was conducted using diagnosis and treatment data of DAA drugs for Hepatitis C patients registered in special disease outpatient reimbursement policy and general outpatient clinic from November 2016 to December 2020, and therapeutic effectiveness data of hepatitis C patients from 2014 to 2020.

#### 2.1.1. Ethnical approval

The study was reviewed and approved by the Ethical Committee of West China Hospital in Sichuan University (reference: 2021-760) and all subjects signed informed consent forms.

### 2.2. Indicators and method of calculation

#### 2.2.1. Diagnostic and therapeutic indicators

Diagnosis and treatment indicators included outpatient visits and outpatient treatments. The registration number and the medical item details occurring at the same time were defined as one visit, and the medical item details that have drug use records were recorded as one treatment. Statistical analysis of the number of monthly visits and treatments from November 2016 to December 2020 were done based on the occurrence of medical details.

#### 2.2.2. Medical cost indicators

The medical cost indicators mainly included the total cost of single outpatient visit, the out-of-pocket cost of single outpatient visit, the cost of of drugs for single outpatient visit, the total cost of all outpatient visits, the out-of-pocket cost of all outpatient visits, and the total cost of drug for all outpatient visits. Since the medical expenses of patients in the City of Chengdu are settled once every 3 months, the cost of single outpatient visit, the out-of-pocket cost of single outpatient visit, and the cost of drugs for single outpatient visit per person from November 2016 to December 2020 were calculated based on the onset of the treatment. The cost of treatment for hepatitis C patients was calculated based on a course of at least 48 weeks for the traditional treatment (interferon peg and ribavirin) and a 12-week course for the DAA treatment. Since patient data was only obtained from one designated hospital, which might include data of patients with incomplete treatment, it was not possible to accurately obtain the cost of each patient's course of treatment. Therefore, for patients using the traditional protocol, the cost of all single visits in 3 months multiplying by 4 was used to calculated the cost of the entire course of treatment, and for patients using the DAA protocol, the cost of all single visits in 3 months approximates to the cost of the entire course of DAA treatment.

#### 2.2.3. DAA drug use indicators

Drug use indicators included the number of DAA drug users and the proportion of DAA programs. Based on the availability of data, the number of monthly usage of DAA drugs from November 2018 to December 2020 and the use of each DAA program were analyzed.

#### 2.2.4. Indicators of therapeutic effect

Therapeutic effect indicators included SVR rate, time to obtain SVR, annal outpatient visits, and annual per capita outpatient interval. The main measure of a patient's therapeutic effectiveness was SVR, i.e., RNA testing is negative 3–6 months after the end of treatment. Patients who had at least one positive HCV-RNA test from 2014 to 2020 were screened in this study, proportion of patients using DAA drugs and patients using traditional protocols who tested negative for RNA 3–6 months after completing a course of treatment were calculated, and the average time needed for positive RNA results to turn negative for patients in each treatment were calculated.

### 2.3. Data statistics and analysis

#### 2.3.1. Descriptive statistical analysis

In this study, descriptive statistical analysis methods were applied to the drug use indicators and treatment effect indicators to analyze the changes in the types of drugs used and the number of users, and the therapeutic effects of patients in different treatments over time.

#### 2.3.2. Interrupted time series analysis

The Interrupted Time Series (ITS) model was used to analyze the diagnostic and medical cost indicators. Measurement data of outcome indicators at multiple time points before and after each policy implementation were collected to compare the instantaneous horizontal changes and trend changes in outcome indicators before and after policy implementation to assess the impact of interventions.

For hepatitis C patients, an outpatient reimbursement policy and a one-line payment policy for state-negotiated drugs were successfully adopted in Chengdu. The outpatient reimbursement policy was implemented in November 2018 in Chengdu as a pilot policy, so the pre-intervention observation period of the special disease outpatient reimbursement policy was set from November 2016 to October 2018, and the observation period after the intervention was set from November 2018 to December 2019. In this policy, the DAA drug costs incurred by outpatients in the treatment process in designated medical institutions are calculated per person, with maximum payment of 43,100 yuan / person and no minimum deductibles; urban medical insurance participants are reimbursed at 80%, and among the urban and rural medicare insurance participants, high-end contributors and low-grade contributors are reimbursed at 70 and 60%, respectively. The scope of hepatitis C reimbursable drugs includes Sophobuvir vipatamivir tablets, Oberpagli Tablets, Dasebvir Sodium Tablets, Albavir Gravevir tablets, Ashurevir Soft Gels, Dalatamivir Hydrochloride Tablets, and Danorevir Sodium Tablets. Medical expenses other than drug costs are paid on a per-item basis and reimbursed in accordance with the special disease outpatient payment policy. The diagnosis and treatment fee is settled by the medical insurance handling agency and the designated medical institution, and the drug fee is settled by the medical insurance handling agency and the drug supply coverage entity. At the end of 2019, three DAA drugs entered the medical insurance directory through the national medical insurance price reduction negotiation, including Albavir Gravivir, Lydipaivir Sophosbuvir, and Sophobuvir vipatamivir. The single-line payment policy covered three DAA drugs was implemented thereby in Chengdu. In this reimbursement policy, there is no differentiation between outpatient and hospitalization, minimum deductible is not set, and it is directly paid by the basic medical insurance in a certain proportion. The drug single-line payment reimbursment ratio of urban medicare insurance participants is 75%, the for high-end and low-grade rural participants is 65 and 55%, respectively, and the maximum single-line payment limit for individuals is 150,000 yuan. Compared with other negotiated drugs that are included in the scope of Class B management, DAA drugs for hepatitis C are given better reimbursement policy. The single-line payment policy was implemented in January 2020, so the observation period after the intervention of the single-line payment policy is set from January 2020 to December 2020. The possible changes in the projection outcome indicators after the implementation of the two policies are shown in Table 1. The effects of policy interventions may take some time to become observant, there may be a lag in the actual analysis process. To avoid mis-estimating the effects of policies, outcome values during the transition period were statistically excluded in this study (13, 14).

**Table 1:** Research indicators and hypothesis.

**First indicators**	**Secondary indicators**	**Possible outcome of classified outpatient payment policy (2018.11)**	**Expecte** **change (2018.11)**	**Possible outcome of including DAA drugs in medical insurance coverage (2020.01)**	**Expected** **change (2020.01)**
Treatment indicators	Number of outpatient visits	Encourages patients to actively seek medical advice	Increase	Encouraged patients to actively seek medical advice	Increase
	Number of outpatient treatment	Encourages patients to actively seek medical treatment	Increase	Encouraged patients to actively seek medical treatment	Increase
Medical expense indicators	Outpatient settlement of total costs	Settlement cost increases temporarily	Increase	Reduce the burden of medical expenses on patients	Decrease
	Out-of-pocket expenses for outpatient settlement	Out-out-pocket expense increases temporarily	Increase	Reduce the burden of out-of-pocket medical expenses	Decrease
	Outpatient settlement of drug costs	Medication expense increases temporarily	Increase	Reduce the burden of drug costs	Decrease
	Total cost of outpatient treatment	No significant change in treatment cost	No change	Reduce the burden of medical expenses on patients throughout the treatment process	Decrease
	Out-of-pocket cost of outpatient treatment	No significant change in out-of-pocket cost	No change	Reduce the burden of out-of-pocket medical expenses throughout the patient's treatment	Decrease
	Outpatient medication costs	No significant change in medication cost	No change	Reduce the burden of drug costs throughout the patient's treatment	Decrease

The statistical model of intermittent time series constructed in this study (15) is as follows:

Y_t_ = β_0_ + β_1_^*^ time_t_ + β_2_^*^ intervention_1t_ + β_3_^*^ time after intervention_1t_ + β_4_^*^ intervention_2t_ + β_5_^*^ time after intervention_2t_ + e_t._

β0 is the horizontal estimate of the baseline outcome index, i.e., the value of the outcome index at *t* = 0.

β1 was the trend estimate of the outcome index values changing with the time variable t before the implementation of the intervention, that is, the baseline slope estimate.

β2 refers to the amount of cross-sectional change in outcome indicators after the implementation of the first intervention policy, reflecting the estimation of the instantaneous level change in the outcome index values resulting from the first intervention policy.

β3 refers to the estimated trend change of outcome indicators resulting from the first policy intervention, that is, the difference between the trend value (slope) of the outcome indicator over time after the implementation of the first intervention and the trend value of the change over time before implementation.

β4 refers to the amount of intercept change in outcome indicators after the implementation of the second intervention policy, reflecting the estimated instantaneous level change in outcome indicator values resulting from the second intervention policy.

β5 refers to the estimated trend change of the outcome indicator resulting from the second policy intervention, that is, the difference between the trend value (slope) of the outcome index over time after the implementation of the second intervention and the trend value of the change over time before the implementation of the second intervention.

et is the error term, which represents some random error in the model that cannot be explained by the above parameters.

In this study, ordinary Least Squares regression models (OLS) was used to perform data analysis, and the Durbin-Watson test and lagrange Multiplier (LM) test were used to test the sequence correlation of the model (16), and the White test was used to perform a heteroscedasticity test for the modeling process (17). Heteroskedasticity and Autocorrelation Consistent Standard Error (HAC) were used to correct for autocorrelation and heteroscedasticity of model data. EViews 8 software was applied for interrupted time series analysis.

## 3. Results

### 3.1. Basic situation

A total of 2,715 hepatitis C cases were collected before and after the implementation of the policy, including 133 special outpatient cases using DAA drugs, and 513 ordinary outpatient cases using DAA drugs. According to the statistical results, November 2018 to February 2019 was a policy transition period, in order to avoid mis-estimating the effect, the data during the transition period were not included in the statistical analysis.

### 3.2. Results of patient visits and treatment analysis

**Figure 1:**
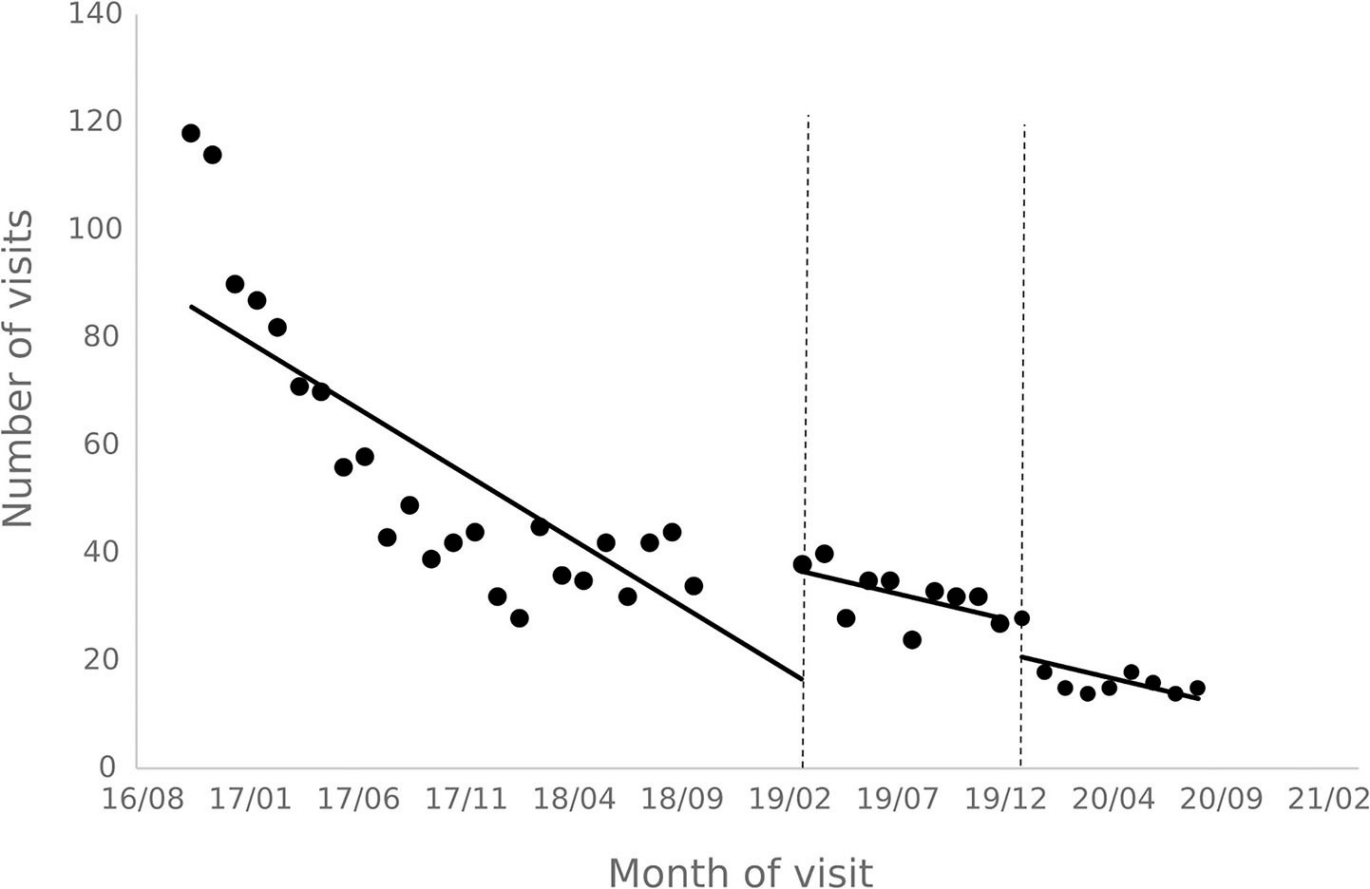
Outpatient visits to patients with hepatitis C.

**Figure 2:**
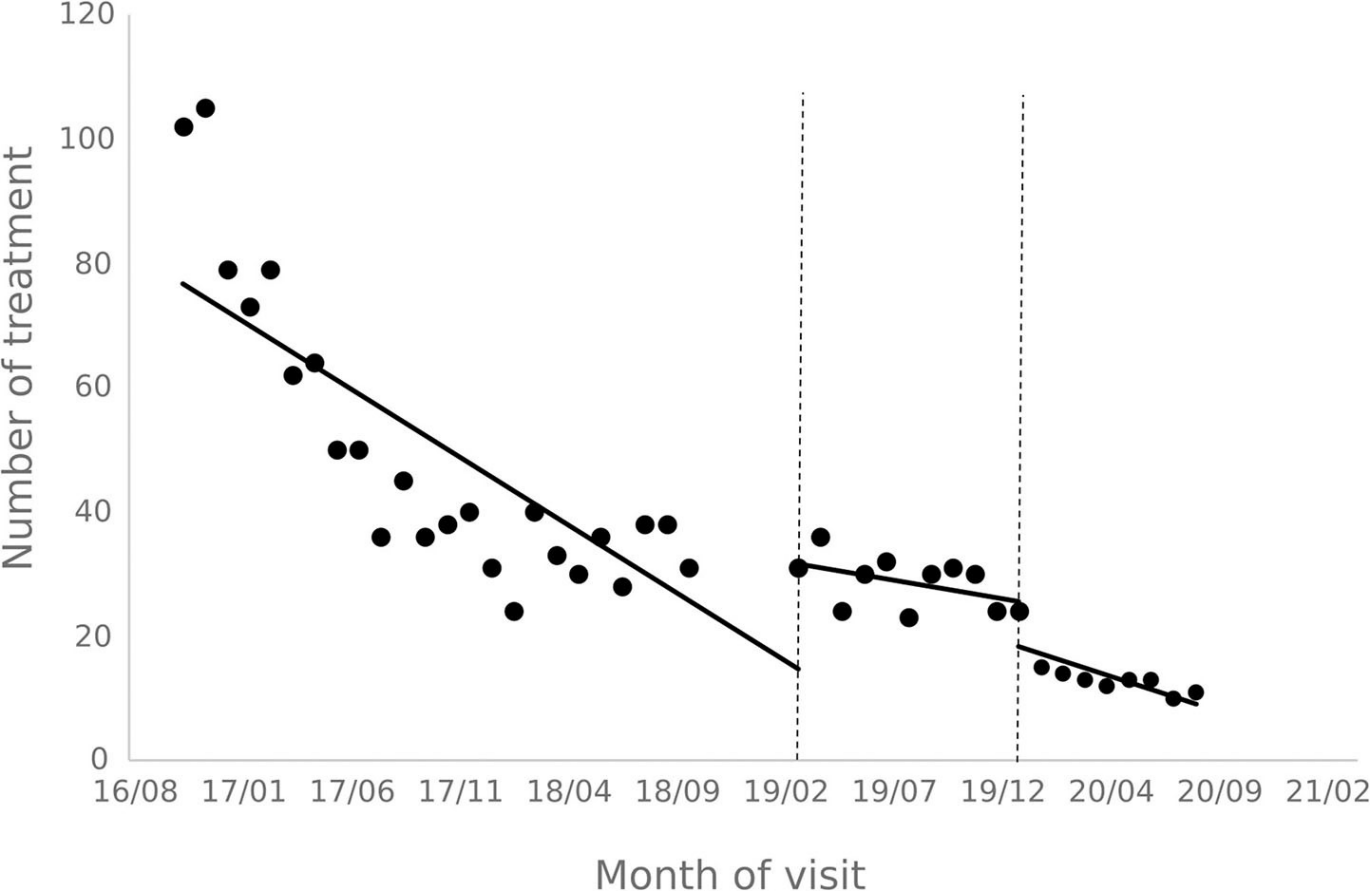
Outpatient treatment of hepatitis C patients.

The results of the interrupted time series analysis of the number of patient visits and treatments of hepatitis C patients are shown in Figures 1, 2; Table 2. The statistical results of the model show that before the implementation of the special disease outpatient reimbursement policy, the number of visits and treatments of hepatitis C patients showed a significant downward trend (*p* < 0.001). After the implementation of the policy, there was a significant instantaneous increase in the number of visits (β2 = 19.290, *p* = 0.003) and the number of treatments (β2 = 14.291, *p* = 0.011); The downward trend in the number of treatments has slowed down from before the implementation of the policy (β3 = 0.395, *p* = 0.048), and there is no statistical difference in the trend of visits (*p* = 0.136). After the one-line payment policy intervention, there was no statistical difference in the level and trend change of the number of visits and treatments (*p* > 0.05).

**Table 2:** Results of intermittent time series model of diagnostic and therapeutic indicators.

**Indicators**	**Baseline intercept (**β**0)**		**Baseline slope (**β**1)**		**Horizontal variation (**β**2)**		**The amount of trend change (**β**3)**		**Horizontal variation (**β**4)**		**The amount of trend change (**β**5)**	
	**Estimated value**	***p-*** **value**	**Estimated value**	***p-*** **value**	**Estimated value**	***p-*** **value**	**Estimated value**	***p-*** **value**	**Estimated value**	***p-*** **value**	**Estimated value**	***p-*** **value**
Number of visits	89.031	0.000	−2.574	0.000	19.290	0.003	0.336	0.136	5.380	0.062	−0.024	0.808
Number of treatments	79.276	0.000	−2.285	0.000	14.291	0.011	0.395	0.048	3.065	0.251	−0.045	0.574

### 3.3. Analysis of patient medical expenses

#### 3.3.1. Outpatients single medical expenses settlement

**Figure 3:**
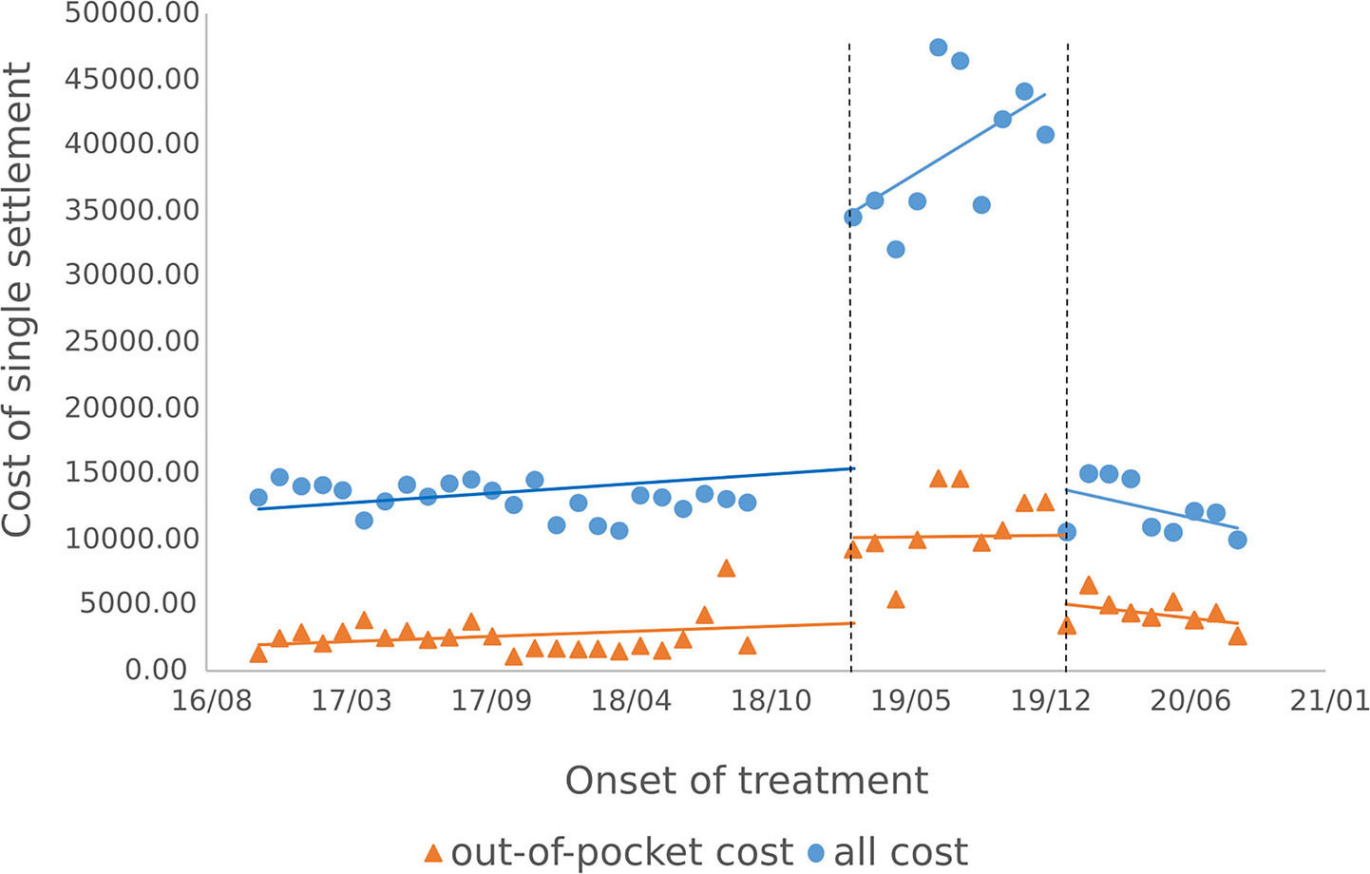
Cost of single settlement for hepatitis C patients.

**Figure 4:**
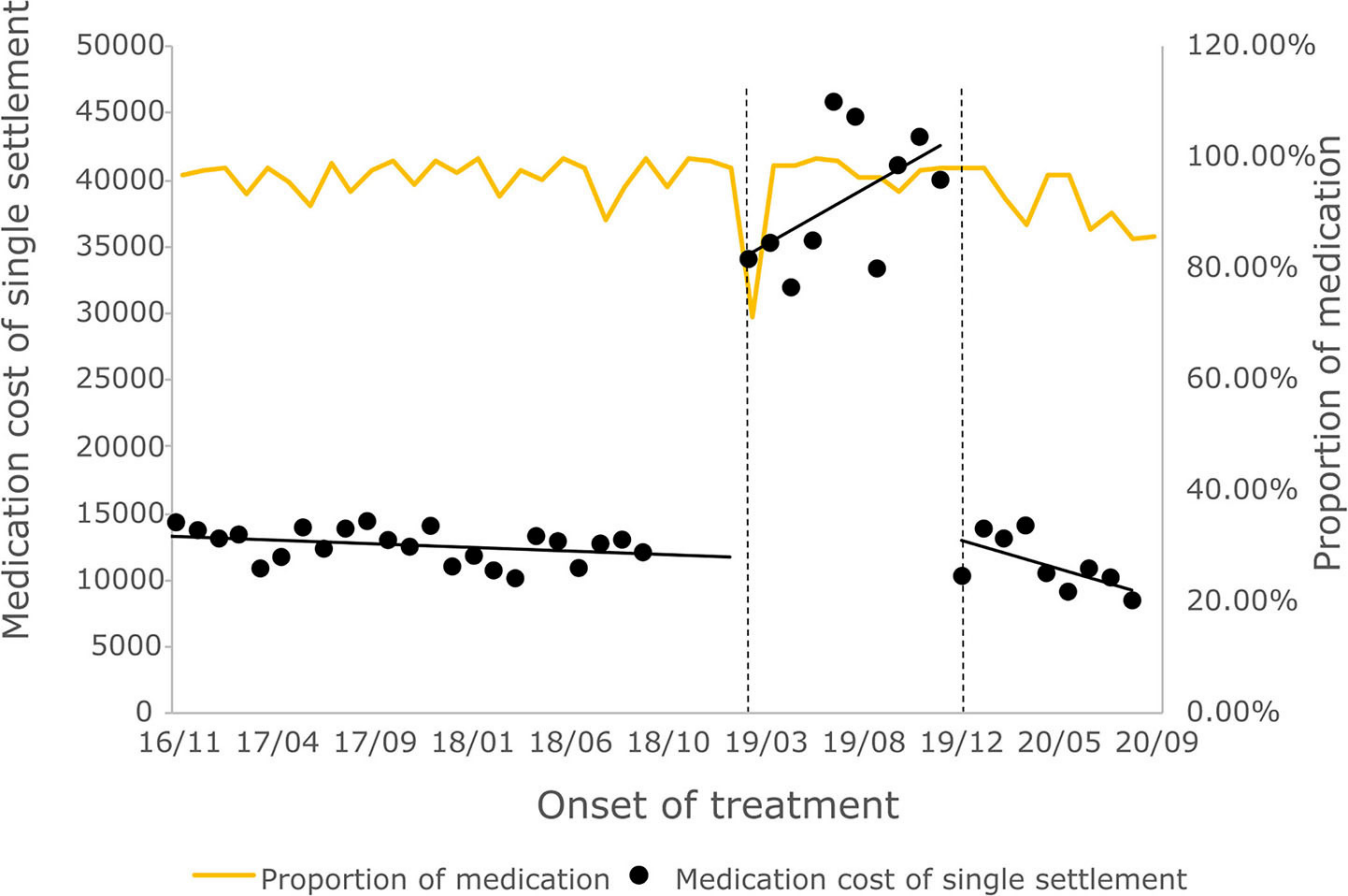
Medication cost of single settlement for hepatitis C patients.

The results of the intermittent time series analysis of the single settlement medical expense of hepatitis C patients are shown in Figures 3, 4; Table 3. The statistical results of the model show that before the implementation of the outpatient reimbursement policy, the total settlement cost, out-of-pocket expenses and drug costs per capita of patients showed a stable trend with time (*p* > 0.05); after the implementation of the special disease outpatient reimbursement policy, the total settlement cost per patient (β2 = 21439.3), out-of-pocket expenses (β2 = 6109.44) and drug costs (β2 = 21889.8) showed a significant instantaneous increase (*p* < 0.001); the trend over time increased significantly compared with before the implementation of the policy (*p* < 0.05). After the implementation of the one-line payment policy, the total cost of patient settlement (β4 = −29,421), out-of-pocket expenses (β4 = −7709.9) and drug costs (β4 = −28,444) have decreased significantly (*p* < 0.001), but the trend has not changed significantly over time (*p* > 0.05).

**Table 3:** Outcome of cost of single settlement in Intermittent time series model.

**Indicators**	**Baseline intercept (**β**0)**		**Baseline slope (**β**1)**		**Horizontal variation (**β**2)**		**The amount of trend change (**β**3)**		**Horizontal variation (**β**4)**		**The amount of trend change (**β**5)**	

	**Estimated value**	***p-*** **value**	**Estimated value**	***p-*** **value**	**Estimated value**	***p-*** **value**	**Estimated value**	***p-*** **value**	**Estimated value**	***p-*** **value**	**Estimated value**	***p-*** **value**
Total single settlment cost	13560.9	0.000	−33.10	0.251	21439.3	0.000	180.727	0.000	−29,421	0.000	8.560	0.806
Out-of-pocket single settlement cost	2198.80	0.000	21.192	0.608	6109.44	0.000	62.161	0.004	−7709.9	0.000	4.285	0.813
Medication cost of single settlement	13297.7	0.000	−51.14	0.112	21889.8	0.000	164.209	0.000	−28,444	0.000	−10.680	0.766

#### 3.3.2. Cost of outpatient course of treatment

**Figure 5:**
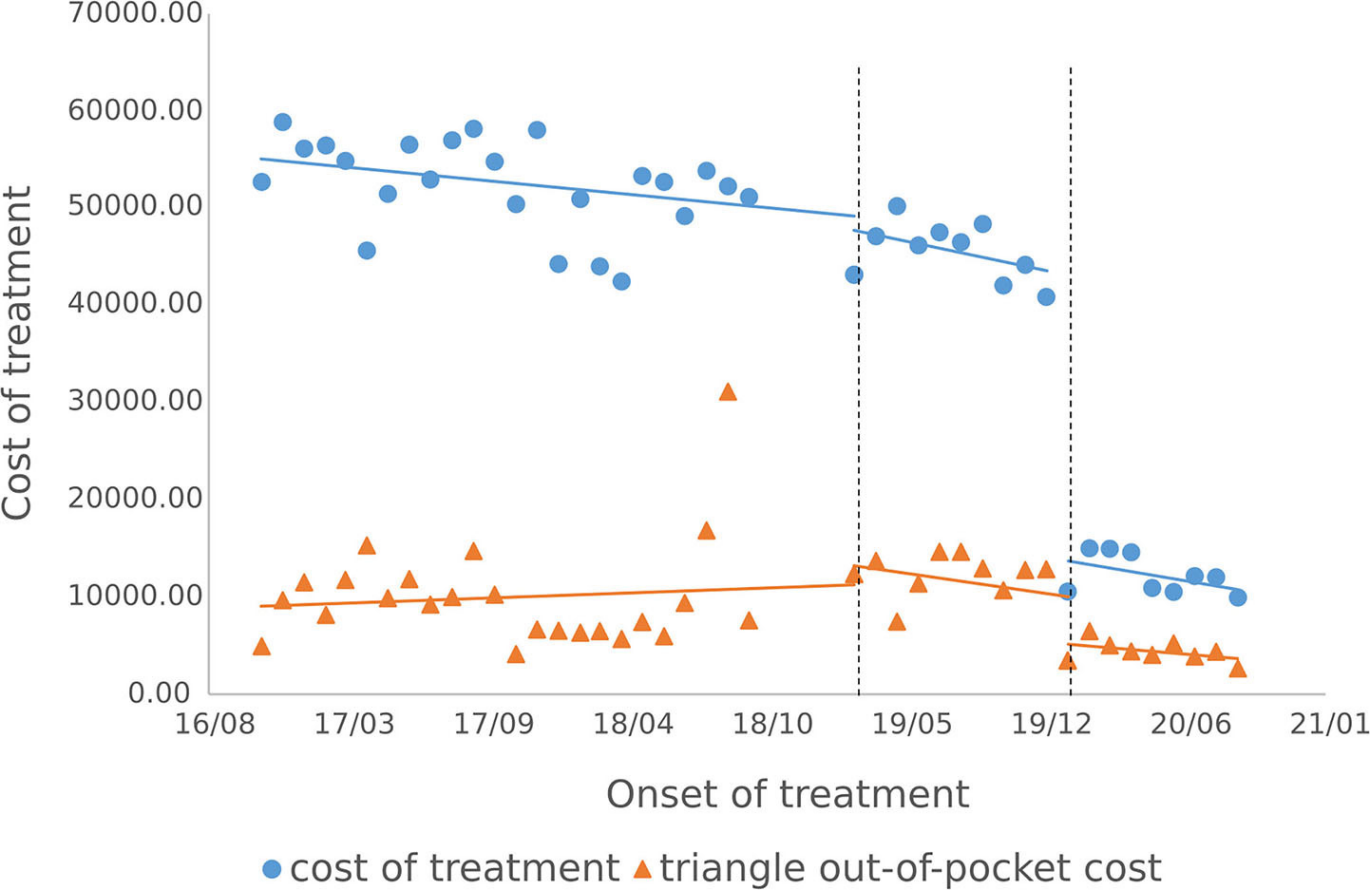
Total treatment expense of hepatitis C patients.

**Figure 6:**
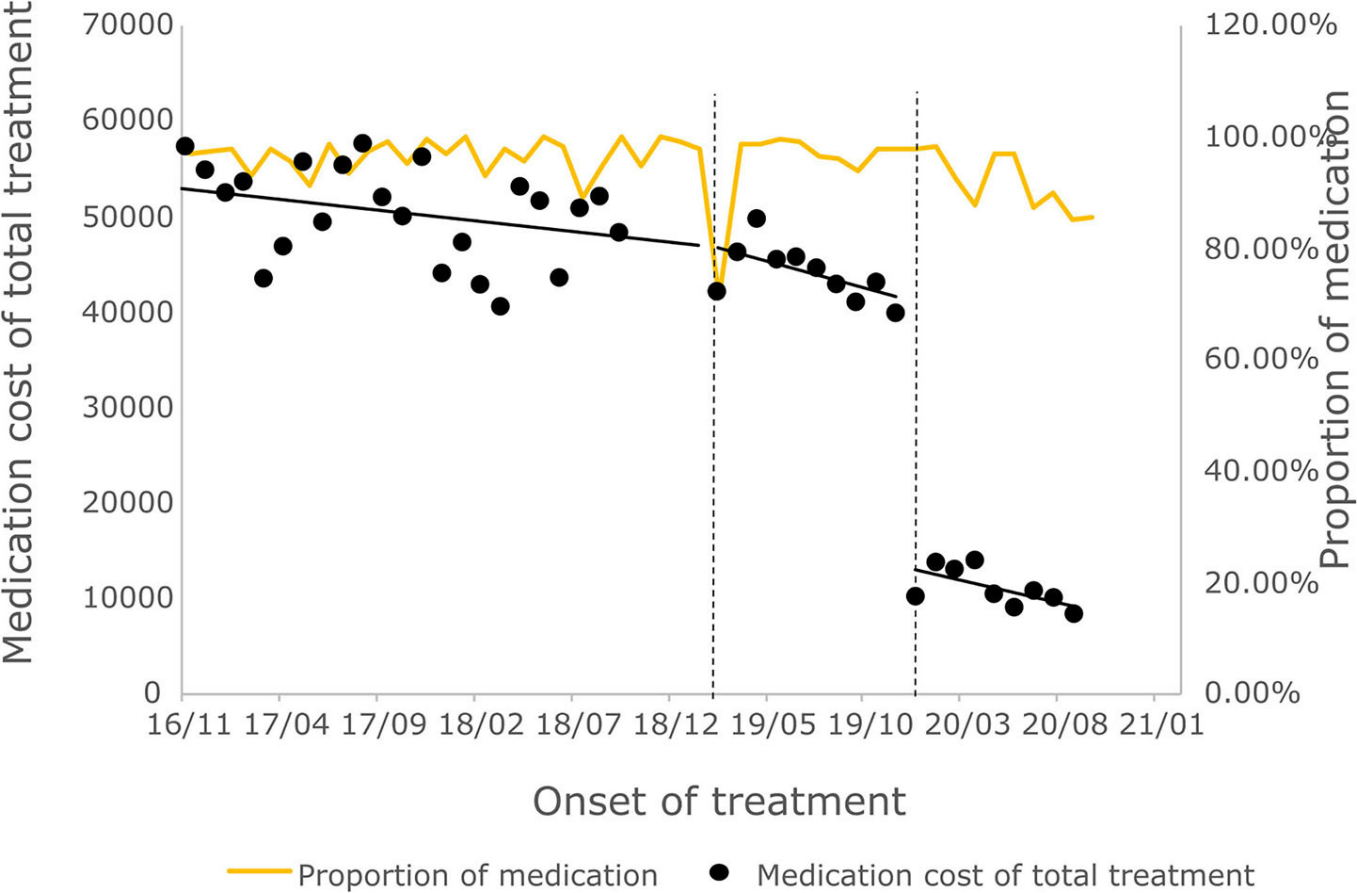
Medication cost and proportion of medication cost in total treatment for hepatitis C patients.

The cost of a single settlement for the above 3 months does not represent the actual medical cost burden of the patients using different treatment options, so the change in the cost of a course of treatment for patients before and after the implementation of the policy is further compared. The results of the intermittent time series analysis during the course of treatment in hepatitis C patients are shown in Figures 5, 6; Table 4. The statistical results of the model show that before the implementation of the outpatient reimbursement policy, the total cost of treatment (β1 = −192.2, *p* = 0.032) and the drug cost (β1 = −258.3, *p* = 0.048) showed a downward trend over time, and the out-of-pocket expenses were relatively stable (*p* > 0.05); after the implementation of the outpatient reimbursement policy, there was no statistical difference in the level of change and trend of change for the three medical expenses (*p* > 0.05). After the implementation of the one-line payment policy, the total cost of the course of treatment (β4 = −33,785), the out-of-pocket cost of the course of treatment (β4 = −8675.7) and the cost of drugs used in treatment (β4 = −32,227) all showed a sharp decline (*p* < 0.001), but the trend did not change significantly over time (*p* > 0.05). Among the outpatient expenses of patients, the proportion of drug costs has a relatively stable trend over time, basically remaining above 90%, as shown in Figures 4, 6.

**Table 4:** Result of treatment cost in in interrupted time series model.

**Indicators**	**Baseline intercept (**β**0)**		**Baseline slope (**β**1)**		**Horizontal variation (**β**2)**		**The amount of trend change (**β**3)**		**Horizontal variation (**β**4)**		**The amount of trend change (**β**5)**	

	**Estimated value**	***p-*** **value**	**Estimated value**	***p-*** **value**	**Estimated value**	***p-*** **value**	**Estimated value**	***p-*** **value**	**Estimated value**	***p-*** **value**	**Estimated value**	***p-*** **value**
Total treatment cost	55111.48	0.000	−192.2	0.032	−4585.1	0.083	47.315	0.524	−33,785	0.000	45.214	0.143
Total out-of-pocket expense	9103.41	0.000	63.511	0.696	1624.08	0.607	−18.252	0.648	−8675.7	0.000	9.0578	0.696
Total medication cost	53970.9	0.000	−258.3	0.048	−2302.8	0.336	38.647	0.639	−32,227	0.000	31.112	0.289

### 3.4. DAA drug usage

#### 3.4.1. Number of people using DAA drugs

**Figure 7:**
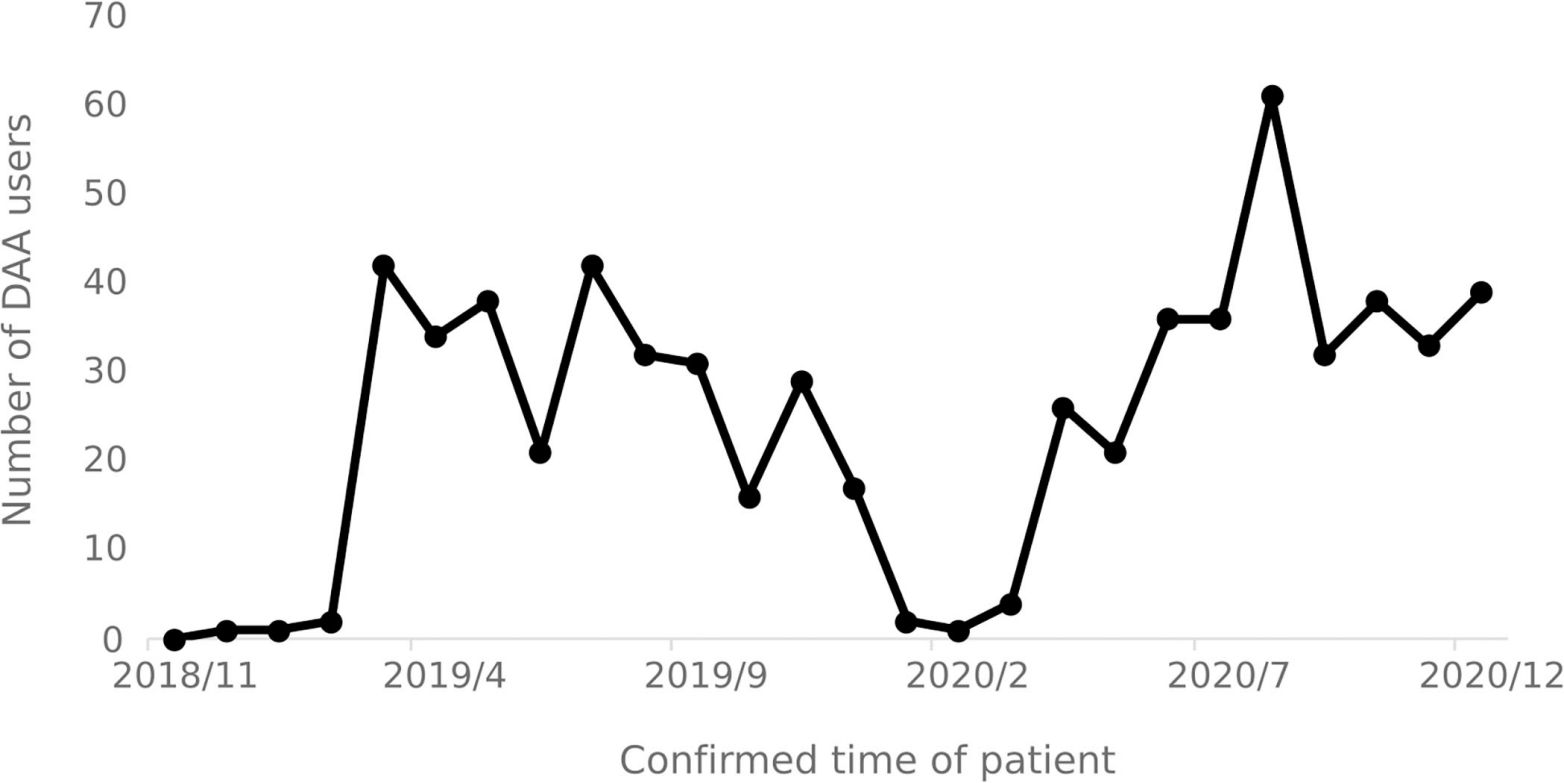
Users of DAA drugs per months.

From November 2018 to December 2020, a total of 635 hepatitis C patients were identified as using DAA drugs. The number of patients using DAA drugs increased significantly in March 2019 and April 2020, respectively, and decreased after September 2020. The number of patients using DAA drugs each month varies over time as shown in Figure 7.

#### 3.4.2. Varieties of DAA drugs used

**Figure 8:**
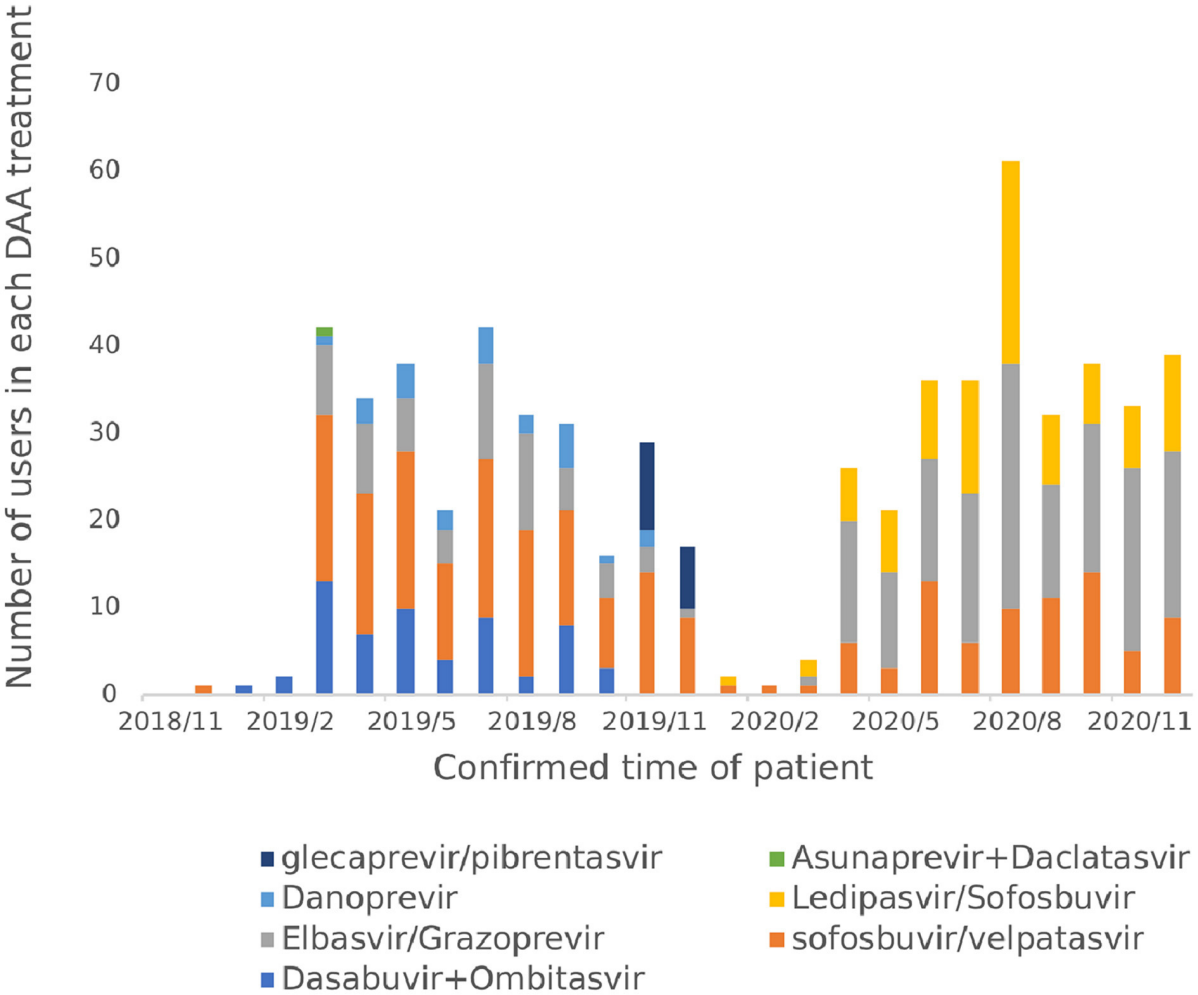
Number of users in each DAA treatment.

From November 2018 to December 2020, a total of 7 DAA regimens were actually used by hepatitis C patients, namely Sofosbuvir and velpatasvir (35.28%), Elbasvir and Grazoprevire (34.02%), Ledipasvir and Sofosbuvir (14.80%), Ombitasvir and Dasabuvir combined (9.29%), Danoprevir Sodium Tablets (3.78%), Glecaprevir and Pibrentasvir (2.68%) and Daclatasvir and Asunaprevir Hydrochloride Tablets in combination (0.16%) as shown in Figure 8. Before the implementation of the single-line payment policy in January 2020, the use of Sofosbuvir and velpatasvir, Dasabuvir combined with Ombitasvir and Elbasvir and Grazoprevir were more frequent; After January 2020, all patients started to only use the three DAA drugs included in the national health insurance list.

### 3.5. Therapeutic effect indicators

According to the results of the special disease outpatient diagnosis and examination of past visits from 2014 to 2020, for patients treated with traditional treatment, the SVR rate was 65.1% (375/576) after the end of the course of treatment, and the SVR rate was 73.8% (425/576) after continuing treatment, and the average RNA negative detection interval was 1.31 years. For patients treated with DAA, the SVR rate was 97.4% (38/39) after the end of the course of treatment, which was significantly higher than that of patients treated with traditional treatment, of which 30.8% (12/39) of patients were consistently positive for RNA testing after conventional treatment, and SVR was achieved after introducing treatment with DAA drugs. The average time to achieve SVR in patients receiving DAA medication was 2.65 months, significantly lower than that of patients treated with traditional treatment.

**Figure 9:**
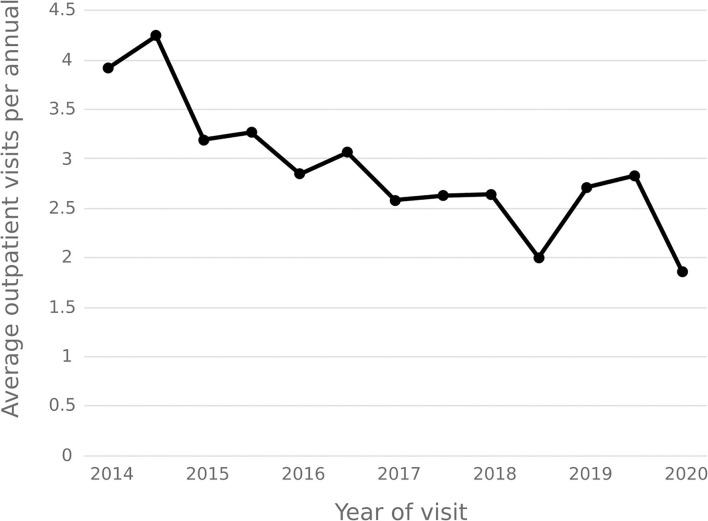
Average outpatient visits of hepatitis C patient per annual.

**Figure 10:**
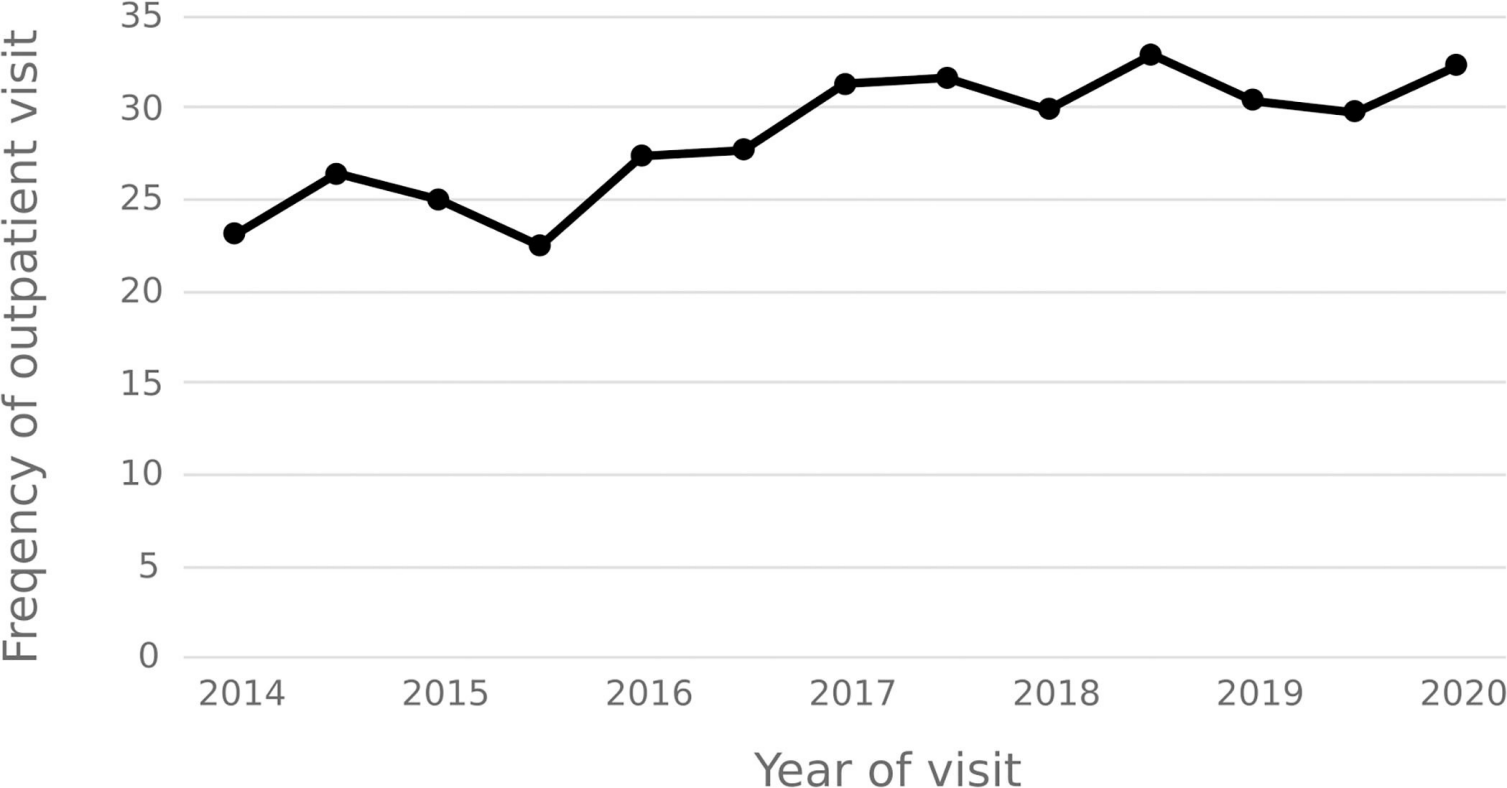
Average frequency of outpatient visit of patient per person.

The statistical results show that the average number of outpatient visits for hepatitis C patients in 2014–2020 shows a downward trend, and the annual per capita outpatient time interval shows an upward trend. In 2020, the number of outpatient visits per capita for hepatitis C patients decreased from 3.92 in 2014 to 1.86, and the annual per capita outpatient interval increased from 23.18 days to 32.36 days. See Figures 9, 10.

## 4. Discussion

### 4.1. The impact of medical insurance reimbursement policy on patient diagnosis and treatment

From the perspective of trend changes, the number of outpatient visits and treatments for hepatitis C has always shown a downward trend, which is inconsistent with the expected assumptions. On the one hand, this is because this study only included the data from one hospital, which may have a certain bias from the overall number of patients. On the other hand, it may be due to the gradual decrease in the number of hepatitis C patients in the context of existing treatment methods and safeguard policies, resulting in a decrease in the number of patients visited, reflecting the positive effect of existing policies on the elimination of hepatitis C.

From the perspective of level change, after the implementation of the special disease outpatient reimbursement policy in Chengdu, the number of outpatient visits and treatment trips have increased instantaneously, and although the number of outpatient treatments still shows a downward trend, its reduction rate has slowed down significantly, indicating that the medical insurance reimbursement policy can significantly motivate hepatitis C patients to seek medical treatment, which is conducive to improving the diagnosis rate and treatment rate of hepatitis C infected people.

### 4.2. The impact of medical insurance reimbursement policy on patients' medical expenses

After the implementation of the special disease outpatient reimbursement policy at the end of 2018, the per capita single settlement cost has increased significantly, of which the drug cost accounts for the largest proportion, This is due to the fact that DAA drugs were involved in the medical insurance per the outpatient reimbursement policy in Chengdu without any price adjustment, which contributed to the significant increase in the outpatient expense. However, due to the implementation of the reimbursement policy, the average increase in OOP per patient is much smaller than the total cost of outpatient clinics. In addition, short term medical expense cannot represent actual medical burden as DAA treatment only lasts for 12 weeks where as traditional pegyneol interferon treatment lasts up to 48 weeks. By further analyzing the change in the treatment costs before and after the implementation of the policy, it was discovered that the total treatment cost and out-of-pocket treatment cost of hepatitis C patients have not changed significantly under outpatient special disease reimbursement policy, and the out-of-pocket costs during the course of treatment have basically remained below 20,000 yuan, which is slightly lower than the relevant results conducted by other research teams using data from the Chengdu Municipal Medical Insurance Bureau (18). The result may due to the fact that the patients included in this study are participants of special disease outpatient reimbursement policy, whose reimbursement ratio for examinations and treatments costs other than drug costs are higher.

Drug price is an important factor that impacts the medical expense of hepatitis patients. Hepatitis C patients are mainly treated with DAA drugs, and the proportion of drug costs has reached more than 90%, accounting for the largest proportion of outpatient medical expenses for hepatitis C patients. In 2019, three DAA drugs were included in the national medical insurance catalog through medical insurance price reduction negotiation, and the unit price of drugs fell by more than 85%. The price reduction of DAA drugs has significantly reduced the average drug cost and drug cost of total treatment of hepatitis C patients, which in turn significantly reduced the total out-of-pocket of a single visit and the total out-of-pocket cost of treatment for hepatitis C patients. The overall results showed that after the implementation of the two policies, the financial burden of hepatitis C patients has been significantly reduced.

### 4.3. Use of DAA drugs after the implementation of the policy

The number of users reveals that there is a lag of 2 to 3 months in the implementation of the special diseases outpatient reimbursement policy and the single-line payment policy, as such implementation efficiency of relevant medical insurance policies in need to be improved. Since April 2020, the number of users of DAA treatment has shown an increasing trend, indicating that the national hepatitis C medical insurance policies have played a role in promoting the promotion and use of DAA treatment. The slight decrease in the number of DAA drug users after September 2020 was due to the overall decrease in the number of infections and the number of hospital visits by hepatitis C.

In terms of treatment options, the WHO guidelines for the diagnosis and treatment of hepatitis C and the guidelines for the prevention and treatment of hepatitis C in China both preferentially recommend the use of pan-genotype for treatment (2, 6), which do not require genotyping before treatment, require less monitoring during treatment, and are more convenient to use and manage than genotype-specific regimens. Sofosbuvir and velpatasvir is the world's first pan-genotype DAA drug that can be used to treat hepatitis C patients with all six genotypes (6). In terms of therapeutic efficacy, a Phase III clinical trial in 38 Asian regions in China, Thailand, Vietnam, Singapore, and Malaysia confirmed high clinical efficacy and safety (19). The European Society for Liver Research (EASL) guidelines, the American Society for the Study of Liver Diseases (AASLD)/American Society of Infectious Diseases (IDSA) guidelines, and the WHO guidelines all recommend Sofosbuvir and velpatasvir as a first-line treatment regimen (2, 7, 11). In November 2018, Sofosbuvir and velpatasvir was the only pan-genotype program in the scope of reimbursable DAA drugs in Chengdu outpatient clinics, and among the DAA drugs included in the national medical insurance list in 2019, the drug was the only non-gene type 1b reimbursable treatment drug. The results of the real-world patient medication study based on the database of a hospital in Sichuan Province also conform to the guidelines and recommendations and relevant medical insurance policies, and the proportion of patients using Sofosbuvir and velpatasvir has always remained high, which is consistent with the results of other studies related to drug use in China (20).

The proportion of patients in the hospital who use genotype-specific protocols is 62%, and drug price is one of the factors influencing the choice of drugs. The price of genotype-specific regimens is lower than that of pan-genotype regimens (6), as such it is still recommended clinically. After December 2019, the patient's medication regimen is concentrated in Sofosbuvir and velpatasvir, Elbasvir and Grazoprevir, and Ledipasvir and Sofosbuvir, because after DAA drugs are included in the national medical insurance list, other drugs other than the three drugs in the medical insurance list are no longer reimbursed, so the use of drugs within the scope of protection is given priority to reduce the patient's out-of-pocket costs, and the choice of drug is consistent with the national medical insurance policy. With the continuous expansion of the variety of DAA drugs included in the scope of reimbursement in the future, clinicians should select appropriate treatment plans for precise treatment according to factors such as patient genotype, disease severity, treatment history, drug price and reimbursement rules, and further improve the therapeutic effect of patients.

### 4.4. Analysis of the therapeutic effect of hepatitis C patients

In this study, the SVR rate of patients using the traditional interferon treatment was only 65.2%, which was slightly lower than that of relevant studies and related statistics at within China and abroad (1, 21): the SVR rate of patients using the DAA drug treatment was 97.4%, which was basically consistent with the statistics of other relevant real-world clinical studies and guidelines (6, 22), further verifying the effectiveness of DAA drugs. Among the patients who were cured, the average treatment time of patients using DAA drugs was 2.65 months, which was only one-sixth of the traditional interferon treatment time. As the treatment time required by patients was significantly reduced, it positively improved patient medication compliance and treatment efficiency of hepatitis C patient. Therefore, the marketing and promotion of DAA drugs will have a clear positive effect on improving the therapeutic effect of hepatitis C and achieving the goal of eliminating hepatitis C.

### 4.5. Limitation

There were a certain limitations in this study. First of all, although Chengdu is the provincial capital with a population second only to Beijing, Shanghai and Chongqing, there are variations in China's hepatitis C medical insurance policies in different cities. It is necessary to carry out real world research involving more cities or at national levels to have a more comprehensive understanding of the implementation effect of hepatitis C medical insurance policies. In addition, considering the availability of data, the use of DAA drugs is only included in the data after the implementation of the special disease outpatient reimbursement policy in November 2018, so it is still impossible to collect data on the number, proportion and cost of DAA drugs before the implementation of the policy. We need to further expand the sample size to evaluate the effect before and after the implementation of the special disease outpatient reimbursement policy.

## 5. Conclusion

This study found that China's hepatitis C medical insurance policy, taking Chengdu as an example, has had a positive effect on the prevention and treatment of hepatitis C and the reduced the disease burden of patients. Firstly, Advanced experience needs to be summarized in a timely manner, the content and methods of hepatitis C medical insurance reimbursement policy needs continuous expansion and optimization, and the policy needs to be actively promoted it other co-ordination regions. At the same time, by establishing of a multi-level insurance system and improving of the “dual-channel” management mechanism and other policies, the implementation of medical insurance for DAA drugs can be further promoted, thus improving the accessibility of the drugs.

## Data availability statement

The original contributions presented in the study are included in the article/supplementary material, further inquiries can be directed to the corresponding authors.

## Ethics statement

The study was reviewed and approved by the Ethical Committee of West China Hospital in Sichuan University (reference: 2021-760) and all subjects signed informed consent forms.

## Author contributions

Sorted the data and wrote the paper: LY and GL. Collected data and statistical analysis: GZ and FH. Managed and supervised the project: FH and HM. Revised and reviewed the paper: HM, WZ, and HQ. All authors contributed to the article and approved the submitted version.
